# Pediatric first aid knowledge and attitudes among staff in the preschools of Shanghai, China

**DOI:** 10.1186/1471-2431-12-121

**Published:** 2012-08-14

**Authors:** Feng Li, Fan Jiang, Xingming Jin, Yulan Qiu, Xiaoming Shen

**Affiliations:** 1 Department of Developmental and Behavioral Pediatrics, Shanghai Pediatric Translational Research Institute, Shanghai Children's Medical Center affiliated Shanghai Jiaotong University School of Medicine, Shanghai Key Laboratory of Children’s Environmental Health, MOE-Shanghai Key Laboratory of Children's Environmental Health, Ministry of Education, China, 1678 Dongfang Rd, Shanghai 200127, China; 2 Department of Children and Adolescents Health Care, Xin Hua Hospital affiliated Shanghai Jiaotong University School of Medicine, Shanghai Key Laboratory of Children's Environmental Health, MOE-Shanghai Key Laboratory of Children's Environmental Health, Ministry of Education, China, 1665 Kongjiang Rd, Shanghai 200092, China; 3 School of Public Health affiliated Shanghai Jiaotong University School of Medicine, 227 South Chongqing Rd, Shanghai 200025, China; 4 Shanghai Institute for Pediatric Research, Xinhua Hospital affiliated Shanghai Jiaotong University School of Medicine, Shanghai Key Laboratory of Children's Environmental Health, MOE-Shanghai Key Laboratory of Children's Environmental Health, Ministry of Education, China, 1665 Kongjiang Rd, Shanghai 200092, China

**Keywords:** First aid, Preschool staff, Knowledge

## Abstract

**Background:**

Unintentional injury remains the leading cause of morbidity and mortality among children worldwide. The aims of this study were to assess a baseline level of first aid knowledge and overall attitudes regarding first aid among staff members in Shanghai preschools.

**Methods:**

A cross-sectional study was carried out among the staff members at selected preschools. A stratified random sampling method was first used to identify suitable subjects. Data were obtained using a multiple-choice questionnaire. A standardized collection of demographics was performed and participants were given the aforementioned questionnaire to indicate knowledge of and attitudes toward first aid.

**Results:**

1067 subjects completed the questionnaire. None of the surveyed employees answered all questions correctly; only 39 individuals (3.7%) achieved passing scores. The relative number of correct answers to specific questions ranged from 16.5% to 90.2%. In particular, subjects lacked knowledge regarding first aid for convulsive seizures (only 16.5% answered correctly), chemical injuries to the eye (23%), inhaled poison (27.6%), and choking and coughing (30.1%). A multiple linear regression analysis showed scores were significantly higher among staff members with more education, those who had received first aid training before or were already healthcare providers, younger employees, and staff members from rural districts. Most employees agreed that giving first aid was helpful; the vast majority felt that it was important and useful for them to learn pediatric first aid.

**Conclusions:**

The level of first-aid knowledge among preschool staffs in Shanghai was low. There is an urgent need to educate staff members regarding first aid practices and the various risk factors relating to specific injuries.

## Background

Injuries and accidents are the leading causes of death in children worldwide [1]. Children are prone to unintentional injuries [2] and are at a higher risk of experiencing injuries, because their bodies are developing and they have not yet learned to be aware both of themselves and various environmental dangers. Because children spend a significant portion of their day in child-care centers, pediatric emergencies such as the exacerbation of existing medical conditions or accidental physical injuries are more likely to occur in those settings [3]. Unintentional injuries, such as falls, bruises, and bumps likewise occur in child-care programs. Schools and playgrounds are the most common location for falls (40.4%), and approximately 39% of reported injuries in child-care settings involve bites [4]. In the United States, annual injury rates range from 0.7 to 5.1 injuries per child [5]. Injury alone accounts for almost one half of all deaths in preschool-aged children in the USA [6,7]. In China, injury accounts for a third of all deaths in children aged 1 to 4 years, and one half of all deaths in children between 5 to 9 years of age [8]. The rate of accidental injury was 10.94% among preschool children in Shanghai; the most common injuries included falls, collisions and extrusions, and sprains [9]. Compared to non-school-based emergency medical service (EMS) incidents, school-based EMS incidents are more often attributable to injury, more often related to a sports activity, and more often result in transport to a medical facility [10]. Most injuries in preschools are require only first aid treatment [11]; therefore, preschools are important locations to focus on the prevention of injuries and diseases in children, because situations requiring first aid are often encountered there. Leila et al. described the first aid used and resulting clinical outcomes of all patients who arrived at a children’s hospital with an acute burn injuries [12]. They found that correct first aid was associated with significantly reduced re-epithelialization time for children with contact injuries; likewise, some positive clinical outcomes were associated with correct first-aid use. This shows there is a need for a higher public awareness of correct first-aid treatments. Administering correct and timely first aid to patients after accidents is vital and can potentially save lives [13,14]. In schools, staffs are often first-aid providers it has become important to determine the current perceptions held by school staff concerning children’s accidents [15].

Trained individuals who are closest to the scene of the accident should administer first aid, first aid training for regulated day care providers may contribute to children's health and safety [16]. A pediatric first aid training program for the staff members at Shanghai preschools has already begun. This program will equip all 35,000 current preschool employees in Shanghai with medical first aid knowledge and skills. Concurrent with the training program, a baseline survey was conducted on the preschool staff members. The goal of the current study was therefore to use questionnaires to evaluate preschool employees' knowledge of and attitudes toward first aid measures for the management of ill and injured children.

## Methods

### Subjects

This study included employees working in preschools in Shanghai, China. In China, no distinction is made between preschools and day care centers. Preschools in China operate on a full-day basis and thus serve as both an educational and childcare institutions. There are a total of 1193 preschools in Shanghai, employing 35,000 teachers, healthcare providers (persons with some medical knowledge; they are generally not doctors), and directors. Shanghai has 18 districts and one county; a stratified random sampling method was used to obtain 1282 subjects. The number of staff members in each district or county was selected based on the proportion of staffs in the district or county. Of the total 35,000 preschool employees in Shanghai, 54.2% work at public schools, 29.1% at private schools, and 16.7% at other types of schools. In each district or county, preschools are classified as public, private, or other. In the district or county, the employees from each preschool category were selected according to the proportion of the types of schools present in the district or county. A local children’s hospital undertook the stratified sampling process. The selected 1282 employees were invited to meet at the children’s hospital with help from the local education authorities. Finally, 1067 of 1282 volunteers participated in this study. A cross-sectional survey was conducted to assess subjects for the first aid management of ill or injured children using a self-filled questionnaire. The participants were not allowed to check any reference materials. All participants provided written informed consent. The institutional review board of Shanghai Children's Medical Center approved this study, and the research was carried out in compliance with the Helsinki Declaration.

### Procedures

The data were collected by questionnaire, which was divided into three sections. Section A focused on demographic information of the participants. Section B was comprised of 37 simple-choice questions on the knowledge of the treatment of common children’s emergencies. The questions were written using a PedFACTs textbook [17] and an instructor's resource manual published by American Academy of Pediatrics [18]. Participants were instructed to select the best answer from a choice of four options. One point was awarded for each correct answer, with no credit given for unanswered questions or an answer of “Not Sure”. Total scores were computed as a sum of each item score and then standardized to a range of 0–100. A score of 80% or greater was required to pass, in accordance with examination guidelines from the American Academy of Pediatrics. Section C addressed attitudes toward first aid, including three questions apiece on attitudes towards learning and also administering first aid. Participants responded using a five-point scale ranging from disagreement to agreement (1 – Completely Disagree; 2 – Partly Disagree; 3 – Neutral; 4 – Partly Agree; and 5 – Completely Agree.). These questions were taken from another first-aid training study [19]. For the percentages of those who agreed, codes 4 and 5 were combined; for percentages of those who disagreed, codes 1 and 2 were combined.

### Data analysis

All data were entered into the Statistical Package for the Social Sciences for Windows (Version 11.0, Chicago, IL, U.S.) for statistical analysis. The results of the questionnaire are expressed as frequency distributions and were computed in percentages. A comparison of the scores based on groups was performed with an analysis of variance or Student's *t* test as needed. A multiple linear regression analysis was performed to assess the independent contributions of factors associated with the knowledge scores: age, district (urban/rural), type of school (public/other), highest level of education (high school and below/college and above), staff category (healthcare providers/teachers), and previous first aid training (received/not). *P* < 0.05 was considered statistically significant for all analyses.

## Results

### Section A: demographic information

A total of 1067 subjects participated in and completed this study. Of the sample group, 0.3% of the subjects were male and 99.7% were female. In our samples, 66.7% of participants were employees at public schools, 21.8% at private schools, and 10.5% at other types of schools. There was no statistically significant difference between our sample and the total preschool staff in Shanghai (*χ*2 =3.848, *P* = 0.146). A total of 69.2% of the group had not taken any first-aid training courses before. The demographic characteristics of the participants are listed in Table 1.

**Table 1:** Demographic characteristic of the staff and average scores of subgroups

**Characteristics**	**N (%)**	**Mean ± SD**	** *t/F* **	** *P* **
District (N = 1067)			18.031	<0.001
Urban	591 (55.4%)	55.3 ± 12.1		
Rural	476 (44.6%)	58.5 ± 12.6		
Age (years) (N = 1067)			19.091	<0.001
≤30	304 (28.5%)	59.4 ± 12.1		
31–40	369 (34.6%)	57.5 ± 12.1		
≥41	394 (36.9%)	53.8 ± 12.3		
Highest education level (N = 1064)			17.643	<0.001
High school or below	278 (26.1%)	54.1 ± 11.6		
College or above	786 (73.7%)	57.7 ± 12.5		
Staff categories (N = 1067)			25.582	<0.001
Healthcare providers	662 (62.0%)	58.2 ± 12.4		
Teachers	405 (38.0%)	54.3 ± 12.1		
Received first aid training^*^ (N = 1065)			14.521	<0.001
No	738 (69.2%)	55.8 ± 12.5		
Yes	327 (30.6%)	58.9 ± 11.9		
Types of school (N = 1067)			9.562	0.002
Public	722 (67.7%)	57.5 ± 12.5		
Others	345 (32.3%)	55.0 ± 12.1		

^*^Those with unknown data for the specified characteristics are excluded from this table.

### Section B: Knowledge

The average scores differed significantly across staff categories. Healthcare providers had higher scores (Table 1). Subjects who had received first-aid training before had a higher level of knowledge than those who had not. The average scores were significantly different among participants from different districts, participants of different ages, participants from different preschools, and participants who had different levels of education (Table 1).

None of participants surveyed answered all questions correctly. An average score of 56.7 ± 12.4 was documented. Only 39 individuals (3.7%) achieved passing scores. Accurate answers to each specific question ranged from 16.5% to 90.2% (Table 2). Subjects especially lacked knowledge regarding first aid for convulsive seizures (only 16.5% aware), chemical injuries to the eye (only 23% aware), inhaled poison (only 27.6% aware), choking and coughing (only 30.1% aware), and bites to the tongue (only 38.8% aware). Only 21% knew to first survey the scene when dealing with an accident. Correct responses regarding first-aid for fainting (41.2% in total, 44.1% of healthcare providers, 36.5% of teachers) and heatstroke (42.1% in total, 41.7% of healthcare providers, 42.7% of teachers) were also low. When faced with cases of inhaled poison, only 27.6% of staff described appropriate first aid. Accurate responses to first aid on other common emergencies are shown in Table 2.

**Table 2:** Relative number of correct responses to study questions

**First aid knowledge**	**Healthcare providers No. (%) (N = 66)**	**Teachers No. (%) (N = 405)**	**Total, No. (%) (N = 1067)**
Percentage of correct responses from <30%			
Convulsive seizures——position the child on his left side first	119 (18.0)	57 (14.1)	176 (16.5)
Survey the scene first in the accidents	143 (21.6)	81 (20.0)	224 (21.0)
Inhaled poison——remove the child from the toxic area first	203 (30.7)	91 (22.5)	294 (27.6)
Chemical injury to eye——put on disposable gloves and flush the chemical from the eye with lukewarm water	158 (23.9)	87 (21.5)	245 (23.0)
Percentage of correct responses from 30% to 60%			
Choking and coughing child——do nothing, except reassure the child and observe the child closely	202 (30.5)	116 (28.6)	318 (30.1)
Caring for a choking child, call EMS after 2 minutes of performing first aid care	253 (38.2)	142 (35.1)	395 (37.0)
Bites to the tongue ——apply pressure with a piece of gauze or cloth to stop the bleeding	296 (44.7)	118 (29.1)	414 (38.8)
Fainting——lay the child on his or her back and loosen any tight clothing	292 (44.1)	148 (36.5)	440 (41.2)
Heatstroke——cool the child immediately and call EMS	276 (41.7)	173 (42.7)	449 (42.1)
reduce infection by flushing the injured area with running water;	289 (43.7)	169 (41.7)	458 (42.9)
before CPR, determine that breathing and coughing are absent	346 (52.3)	211 (52.1)	557 (52.2)
Insect stings——move the child to a safe area and remove any stingers	387 (58.5)	212 (52.3)	599 (56.1)
Nosebleeds——pinch the soft parts of the nose and press against the bones of the face	423 (63.9)	211 (52.1)	634 (59.4)
Penetrating injury to eye——call EMS	408 (61.6)	230 (56.8)	638 (59.8)
Percentage of correct responses >60%			
Punctures——Soak the wound in clean water	455 (61.2)	250 (61.7)	655 (61.4)
Swallowed poison——remove traces of the poisonous from the child’s mouth first and then call EMS	444 (67.1)	270 (66.7)	714 (66.9)
Bleeding——place firm, direct pressure on the wound	472 (71.3)	250 (61.7)	722 (67.7)
Dog bites——care for the wound and check with animal control officer	496 (74.9)	248 (61.2)	744 (69.7)
Spinal injury——avoid moving the child at all, and keep the neck and back aligned	524 (79.2)	327 (80.7)	851 (79.8)
Burns——place the burned area in cool water	552 (83.4)	318 (78.5)	870 (81.5)
Electrical burns——turn off the power source if possible and call EMS	554 (82.2)	335 (82.7)	879 (82.4)
Bone injury——rest and call EMS	562 (84.9)	341 (84.2)	903 (84.6)
Keeping dangerous materials in an inaccessible place and locked up	605 (91.4)	353 (87.2)	958 (89.8)
Swelling——apply cold, then wrap and elevate the injured body part	614 (92.7)	348 (85.9)	962 (90.2)

Results of the multiple linear regression analysis showed the knowledge score to be significantly higher among staff who had higher education levels (*t* = 2.069, *P* = 0.039), who were from rural districts (*t* = −3.785, *P* < 0.001), who had received first aid training before (*t* = 2.506, *P* = 0.012), those who were already healthcare providers (*t* = 4.546, *P* < 0.001), and younger personnel (*t* = −4.185, *P* < 0.001) (Table 3).

**Table 3:** Multiple regression analysis of factors associated with score of knowledge

	**Beta**	**Standard error**	** *t* **	** *P* -value**
College education or above	0.766	0.370	2.069	0.039
Urban districts	−1.054	0.278	−3.785	<0.001
Received first aid training before	0.790	0.315	2.506	0.012
Working at a public school	0.287	0.311	0.921	0.357
Age	−0.781	0.187	−4.185	<0.001
Healthcare providers	1.382	0.304	4.546	<0.001

### Section C: Attitudes

For attitudes towards administering first aid, the majority felt positive toward providing first aid. Regarding attitudes towards learning first aid, the vast majority felt that it was important and useful skill for them to learn (Table 4).

**Table 4:** Pediatric first aid attitudes of staff

**Statement (figures are expressed in percentages)**	N^*^	**Disagree**	**Neutral**	**Agree**
**Attitudes towards giving first aid**				
That I should give first aid is fair	1037	2.1	3.8	94.1
That I should give first aid is unpleasant	1027	86.7	4.9	8.4
That I should give first aid is very good	1028	5.4	7.4	87.2
**Attitudes towards learning first aid**				
It is good for me to learn first aid	1039	0.6	1.2	98.2
It is useful for me to learn first aid	1040	0.4	0.3	99.3
It is important for me to learn first aid	1043	0.5	0.7	98.8

^*^N denotes the number with valid answers (i.e. excludes those who left blanks or indecipherable answers).

## Discussion

The results of our study indicate overall staff knowledge of first aid to be lacking, evidenced by the low but visible frequency of incorrect responses to common illnesses and injuries. The American Academy of Pediatrics has set 80% as the passing level in its written knowledge exam of pediatric first aid training for caregivers and teachers. According to that criterion, only 3.7% of the surveyed teachers had an adequate knowledge of first aid. Questions related to splinters (incorrect rate: 61.4%), nosebleeds (59.4%), Insect stings (56.1%), fainting (41.2%), reducing infection by flushing the injured area with running water (42.9%), heatstroke (42.1%), bites to the tongue (38.8%), inhaled poison (27.6%), and chemical injury to the eye (23.0%) were most often answered incorrectly. With regard to a child swallowing poison, if the victim vomits, the insulting substance may cause more damage as it passes through the esophagus a second time. Induction of emesis is wrong and harmful [20], but many of the study participants thought it appropriate. In cases of poisoning, 33% of the staff members in our study would not take both the child and the poison bottle directly to the hospital. In the present study, the rate of correct answers to questions about convulsive seizures was lowest; only 16.5% of responders would act to first protect the head. Similarly, Dantas in Brazil demonstrated that teachers’ knowledge about the clinical characteristics and initial procedures to attend a person during a seizure was also unsatisfactory [21]. Fewer than 13% of them knew to protect the head. Childhood encounters with bees, wasps, yellow jackets, hornets, and fire ants can be a natural consequence of children's curiosity and exploration. In most cases, insect stings do not require medical attention. However, a severe allergic reaction to an insect sting can occur very quickly, without warning, and can be life-threatening. Insect bites, especially bee stings, can be fatal in short periods of time if the injured person has an extreme sensitivity and is not given first aid immediately [17]. Mild allergic reactions include hives and swelling, but death can occur from edema of the respiratory system [22,23]. This study determined that 43.9% employees did not know to remove bee stingers immediately, and only 56.1% of employees in the study knew to move the child to a safe area and remove any stingers. With regard to heatstroke, 42.1% of responders knew to cool the child immediately and call EMS. Heatstroke can develop suddenly, and an infant or child with heatstroke will have a body temperature of 106 °F or higher. Once the sweat glands' ability to produce sweat is exhausted, the skin may be dry and hot. If the body temperature does not go down, brain damage and death can occur [17]. In cases of choking and coughing, the number of incorrect responses was disturbing (30.1%). The course of action for dealing with a casualty who is choking but also breathing is to allow him or her to continue coughing, because they still have a clear airway [22,24]. An infant or child with a partially blocked airway continues to breathe, but will usually be coughing. Coughing is the body's way of removing what feels like a foreign object. When there is an object in the airway, forceful coughing is more effective than anything anyone else can do to get the object to move up and out of airway [17]. But most responders indicated that they would clap the child's back. Prior studies regarding the knowledge of first aid knowledge among preschool staffs have been scarce. In the U.S., Gagliardi et al. has indicated that most teachers are deficient in knowledge of emergency care and basic life support modalities [25]. A study conducted in Turkey evaluating the first aid knowledge and attitudes of 312 primary school teachers showed that most of the teachers lacked accurate knowledge about first aid [22]. Physical education teachers at schools in Ireland also showed poor knowledge of how to treat children during emergencies [23].

Injury is a common cause of morbidity and mortality in children. Prompt, appropriate treatment can help decrease this morbidity and mortality. Because children spend the majority of their day in school, teachers should be proficient in basic first-aid skills. In this study, only 30.6% of the study participants had ever received first-aid training before; however, they only had been trained in cardiopulmonary resuscitation and not in the treatment of common children’s injuries. The results of a study carried out by Gagliardi on the extent of training and emergency care knowledge of public school teachers in the U.S. indicated that one-third of surveyed teachers had no specific training in first aid, and 40% had never been trained in cardiopulmonary resuscitation [25]. This is also in keeping with other studies from North America, which found that 30% of teachers had no specific training in first aid, and 40% had never been trained in CPR [26,27]. One U.S. study found that only 75% of child care center administrators surveyed reported that first aid training was required for their staff, and only 15% of child care centers serving children with special care needs required its staff to be CPR-certified [3]. In the U.S., approximately half of all child care centers do not have specific written procedures for urgent medical emergencies, such as severe bleeding, unresponsiveness, poisoning, shock, heart or circulation failure, seizures, head injuries, anaphylaxis, or allergic reactions [3]. The American Academy of Pediatrics recommends that at least one staff member who has successfully completed training in pediatric first aid be in attendance at all times and in all places where children are present. Injury studies conducted at childcare centers have demonstrated that the most common injuries are minor and that severe injuries requiring medical attention comprise only 1% to 7% of the total injuries [28,29]. The most common injuries in childcare centers reported in the literature include fractures, lacerations, contusions, and dental injuries [30,31]. One study reported that 84% of injuries occurring in the child care settings required first aid treatment [11]. More effort should be made to increase first aid and CPR training for childcare center staff members, because many emergencies can be managed with these lifesaving skills. Our results clearly demonstrate there are grave deficiencies in the provision of first aid for children among preschool staffs in Shanghai. The lack of formal and effective emergency care training in teacher preparation programs coupled with a lack of continuing education requirements is one possible explanation for these results [25]. Using data from the present study, a comprehensive campaign should be planned for Shanghai.

The current findings show that healthcare providers scored very high on the questionnaire, which is likely related to the fact that healthcare providers by definition have pre-existing medical knowledge. Younger employees also scored higher, which may be due to younger employees frequently possessing higher educational levels and having already been exposed to newer knowledge regarding first aid. Performing proper first aid is a fairly complex set of tasks. Knowledge is necessary but not sufficient. While the attitudes towards giving first aid is assumed to be of direct importance to performance in first-aid situations, the attitudes towards learning first aid may be of greater, if indirect importance [19]. Our survey showed that most teachers’ attitudes towards giving or learning first aid were positive, but it remains necessary to increase their actual first aid knowledge and skills. Improvement in child care centers' preparedness to respond to emergencies and disasters should include maintaining the immediate availability of potentially life-saving medications and ensuring that all child care center employees are trained in first aid and, where appropriate, CPR [3]. Pediatricians should take an active role in training first responders in pediatric assessment and CPR, and assist preschools in developing disaster plans. Teaching first aid offers an opportunity to educate childcare providers and teachers about risk factors for specific injuries. Identification and actions taken to reduce risk delivered alongside first aid training may reduce the overall rate of child injury.

This study has several limitations. The primary limitation is that the investigation of 1067 employees in Shanghai is not representative of other parts of China, largely because there are significant socio-economic disparities between China’s western and eastern provinces; Shanghai is one of the most developed cities in China. Similar studies conducted throughout the country are necessary. Our study was also limited in that it did not evaluate staff members' skills in implementing first aid. This was because most of our participants had none. First aid knowledge alone does not ensure proper conduct during an emergency.

## Conclusions

Although this study shows that the level of first aid knowledge among personnel who care for children was low, it also shows that they are interested in obtaining proper training. There is an urgent need to educate preschool staffs about first aid practices and the risk factors related to specific injuries. We would recommend that pediatric first aid training be made more widely available to the preschool staffs.

## Abbreviations

EMS, Emergency medical services; PedFACTs, Pediatric first aid for caregivers and teachers; SD, Standard deviation; No, Number.

## Competing interests

The authors have no conflicts of interest.

## Authors’ contributions

FJ and XMS conceived and designed the study, coordinated data collection and reviewed the manuscript. FL carried out the study, collected data, analyzed the data, and drafted the manuscript. FJ and XMJ conducted the field work. All authors read and approved the final manuscript for publication.

## Pre-publication history

The pre-publication history for this paper can be accessed here:

http://www.biomedcentral.com/1471-2431/12/121/prepub
